# Supplementary material for: Host ecology regulates interspecies recombination in bacteria of the genus Campylobacter
Source: eLife. 2022 Feb 22;11:e73552. doi: 10.7554/eLife.73552 (PMC8912921; doi:10.7554/eLife.73552)
Supplement: Transparent reporting form [file elife-73552-transrepform1.docx]

***eLife’s* transparent reporting form**

We encourage authors to provide detailed information *within their submission* to facilitate the interpretation and replication of experiments. Authors can upload supporting documentation to indicate the use of appropriate reporting guidelines for health-related research (see [EQUATOR Network](http://www.equator-network.org/%20)), life science research (see the [BioSharing Information Resource](https://biosharing.org/)), or the [ARRIVE guidelines](http://www.plosbiology.org/article/info:doi/10.1371/journal.pbio.1000412) for reporting work involving animal research. Where applicable, authors should refer to any relevant reporting standards documents in this form.

If you have any questions, please consult our Journal Policies and/or contact us: [editorial@elifesciences.org](mailto:editorial@elifesciences.org).

**Sample-size estimation**

- You should state whether an appropriate sample size was computed when the study was being designed
- You should state the statistical method of sample size computation and any required assumptions
- If no explicit power analysis was used, you should describe how you decided what sample (replicate) size (number) to use

Please outline where this information can be found within the submission (e.g., sections or figure legends), or explain why this information doesn’t apply to your submission:

We used all available *Campylobacter* genomes. For specific analysis a subset of genomes was used. For the donor-recipient analysis, each combination of groups was selected to compare the extent of interspecies recombination into the recipient genomes. Each donor group consisted of 8 isolates to avoid influence of difference in sample size on estimation of the extent of interspecies recombination. Each recipient group included at least 4 isolates. This is also described in the methods section (lines 474-478).

**Replicates**

- You should report how often each experiment was performed
- You should include a definition of biological versus technical replication
- The data obtained should be provided and sufficient information should be provided to indicate the number of independent biological and/or technical replicates
- If you encountered any outliers, you should describe how these were handled
- Criteria for exclusion/inclusion of data should be clearly stated
- High-throughput sequence data should be uploaded before submission, with a private link for reviewers provided (these are available from both GEO and ArrayExpress)

Please outline where this information can be found within the submission (e.g., sections or figure legends), or explain why this information doesn’t apply to your submission:

Genomes sequenced as part of other studies are archived on the SRA associated with BioProject accessions. Additional genomes were also downloaded from NCBI and pubMLST (http://pubmlst.org/campylobacter). Contiguous assemblies of all genome sequences compared are available at the public data repository Figshare (doi: 10.6084/m9.figshare.15061017) and individual project and accession numbers can be found in Supplementary Table 1. A data availability statement is provided in the manuscript (lines 495-502).

**Statistical reporting**

- Statistical analysis methods should be described and justified
- Raw data should be presented in figures whenever informative to do so (typically when N per group is less than 10)
- For each experiment, you should identify the statistical tests used, exact values of N, definitions of center, methods of multiple test correction, and dispersion and precision measures (e.g., mean, median, SD, SEM, confidence intervals; and, for the major substantive results, a measure of effect size (e.g., Pearson's r, Cohen's d)
- Report exact p-values wherever possible alongside the summary statistics and 95% confidence intervals. These should be reported for all key questions and not only when the p-value is less than 0.05.

Please outline where this information can be found within the submission (e.g., sections or figure legends), or explain why this information doesn’t apply to your submission:

Recombining Single Nucleotide Polymorphisms (SNPs) were selected based on their copying probability from the donor group genome to the recipient as inferred by Chromosome Painting analysis. Those that had >90% copying probability were considered in downstream analysis. This is described in the text (lines 484-486).

(For large datasets, or papers with a very large number of statistical tests, you may upload a single table file with tests, Ns, etc., with reference to sections in the manuscript.)

**Group allocation**

- Indicate how samples were allocated into experimental groups (in the case of clinical studies, please specify allocation to treatment method); if randomization was used, please also state if restricted randomization was applied
- Indicate if masking was used during group allocation, data collection and/or data analysis

Please outline where this information can be found within the submission (e.g., sections or figure legends), or explain why this information doesn’t apply to your submission:

The donor and recipient groups were selected based on the host and source from which each isolate was sampled. We excluded samples that represented spillover events and not true host segregated populations. This is described in the methods (lines 474-476).

**Additional data files (“source data”)**

- We encourage you to upload relevant additional data files, such as numerical data that are represented as a graph in a figure, or as a summary table
- Where provided, these should be in the most useful format, and they can be uploaded as “Source data” files linked to a main figure or table
- Include model definition files including the full list of parameters used
- Include code used for data analysis (e.g., R, MatLab)
- Avoid stating that data files are “available upon request”

Please indicate the figures or tables for which source data files have been provided:

All additional supplementary files, figures and tables are provided in the full submission. All genome sequences are available at the public data repository Figshare (doi: 10.6084/m9.figshare.15061017) and individual project and accession numbers can be found in Supplementary Table 1. A data availability statement is provided in the manuscript (lines 495-502).
